# Expiratory-gated Transcutaneous Auricular Vagus Nerve Stimulation (taVNS) does not Further Augment Heart Rate Variability During Slow Breathing at 0.1 Hz

**DOI:** 10.1007/s10484-023-09584-4

**Published:** 2023-03-15

**Authors:** Mikołaj Tytus Szulczewski, Martina D’Agostini, Ilse Van Diest

**Affiliations:** 1 Institute of Psychology, Polish Academy of Sciences, Stefana Jaracza 1, Warsaw, 00-378 Poland; 2 Research Group Health Psychology, Faculty of Psychology and Educational Sciences, KU Leuven, Leuven, Belgium

**Keywords:** Slow breathing, Breathing at 0.1 Hz, Transcutaneous auricular vagus nerve stimulation (taVNS), Heart rate variability (HRV)

## Abstract

As cardiac vagal control is a hallmark of good health and self-regulatory capacity, researchers are seeking ways to increase vagally mediated heart rate variability (vmHRV) in an accessible and non-invasive way. Findings with transcutaneous auricular vagus nerve stimulation (taVNS) have been disappointing in this respect, as its effects on vmHRV are inconsistent at best. It has been speculated that combining taVNS with other established ways to increase vmHRV may produce synergistic effects. To test this idea, the present study combined taVNS with slow breathing in a cross-over design. A total of 22 participants took part in two sessions of breathing at 6 breaths/min: once combined with taVNS, and once combined with sham stimulation. Electrical stimulation (100 Hz, 400 µs) was applied during expiration, either to the tragus and cavum conchae (taVNS) or to the earlobe (sham). ECG was recorded during baseline, 20-minutes of stimulation, and the recovery period. Frequentist and Bayesian analyses showed no effect of taVNS (in comparison to sham stimulation) on the root mean square of successive differences between normal heartbeats, mean inter-beat interval, or spectral power of heart rate variability at a breathing frequency of 0.1 Hz. These findings suggest that expiratory-gated taVNS combined with the stimulation parameters examined here does not produce acute effects on vmHRV during slow breathing.

## Introduction

Non-invasive electrical stimulation of the auricular branch of the vagus nerve (transcutaneous auricular vagus nerve stimulation; taVNS) has recently been the subject of interest for many researchers due to its potential to mimic the effects of invasive VNS without the need for surgical procedures. Researchers have speculated that taVNS could function as a potential add-on treatment in chronic pain management (Chakravarthy et al., 2015; Frangos et al., 2017), cardiovascular diseases (Jiang et al., 2020), and depressive disorders, among others, and preliminary tests of this have been carried out. Because decreased cardiac vagal control is a hallmark of all of those conditions (Kishi, 2012; Koch et al., 2019; Tracy et al., 2016), many researchers have considered it a promising transdiagnostic treatment target. High vagal cardiac control measured with the use of analysis of heart rate variability (HRV; for a description of the use of HRV in the assessment of vagal control of the heart see: Pumprla et al., 2002) is related to better mental and physical health among both ill and healthy individuals (Jarczok et al., 2015; Perna et al., 2020), therefore methods targeting vagally mediated HRV (vmHRV) are receiving increasing attention as a potential health-promoting intervention (e.g., HRV biofeedback; Lehrer et al., 2020). However, to date, studies on the effects of taVNS on vagal control of the heart have provided inconsistent findings (for a review, see Burger et al., 2020). A recent meta-analysis showed evidence for the lack of the effects of acute administration of taVNS on vmHRV among healthy individuals (Wolf et al., 2021). Recently. it has been suggested that taVNS can be combined with a controlled decrease of respiratory rate (*slow breathing*) to enhance activation of vagal afferents and the effects of slow breathing on vagal control of the cardiovascular system (Frøkjaer et al., 2016; Szulczewski, 2022).

Autonomic control of the cardiovascular system is one of the main targets for interventions that are based on slow breathing and a large increase in vmHRV is a well-established effect of slow breathing (Lehrer et al., 2020; Lehrer & Gevirtz, 2014; Russo et al., 2017; Song & Lehrer, 2003; Zou et al., 2017). Slow breathing activates sensory vagal pathways from baroreceptors and respiratory mechanoreceptors (Noble & Hochman, 2019). Therefore, it can be hypothesized that during activation of cardiorespiratory vagal afferents by slow breathing, concurrent stimulation of the auricular branch of the vagus nerve may produce synergistic effects and result in further augmentation of HRV. To date, four studies have investigated the effects of taVNS combined with slow breathing on autonomic control of the heart indexed by vmHRV (Frøkjaer et al., 2016; Juel et al., 2017; Keute et al., 2021). However, results are inconsistent and the study designs limit the conclusions that can be drawn about the effects of taVNS and slow breathing on vmHRV and potential synergistic effects between both methods of vagal stimulation.

The first two studies that combined slow breathing with taVNS reported increased vagal control of the heart during the intervention (Frøkjaer et al., 2016; Juel et al., 2017). Both studies compared taVNS combined with slow breathing only to a control condition with sham stimulation combined with a breath counting task during spontaneous breathing. Because slow breathing was performed only in an experimental condition, the observed cardiac effects may be exclusively driven by slow breathing. Therefore, it is impossible to make inferences about additive or synergistic effects of taVNS and slow breathing from these studies. The third study that combined taVNS with slow breathing employed more conditions, and participants performed the slow breathing task during all of them (Keute et al., 2021). The study showed an increase in vmHRV in the condition with taVNS combined with slow breathing. In the control condition, participants also performed a slow breathing task and electrodes were attached to regions innervated by the vagus nerve, but the stimulator was switched off. In this study, slow breathing was used to avoid confounding effects of variation in respiration during the measurement of vmHRV; therefore increased vmHRV was interpreted as an enhancement of vagal output by taVNS, and the role of potential synergy between slow breathing and taVNS was not taken into consideration. By itself, taVNS has rarely been observed to produce an increase in primary indices of vagally-mediated vmHRV; therefore one of the interpretations of this finding is that the observed increase in vagal heart control during taVNS was a result of the synergy between taVNS and slow breathing. However, because electric stimulation was absent in the control condition, the larger vmHRV can be also interpreted as a result of the cardiovascular response to electrical stimulation at the frequency of 0.1 Hz. This is the frequency of the naturally occurring oscillations in the cardiovascular system that are generated by baroreflex activity (the so-called Meyer wave; Julien, 2006). Previous studies showed that a variety of stimuli, such as emotional pictures (Vaschillo et al., 2008), rhythmic muscle tension (Lehrer et al., 2009; Vaschillo et al., 2011), and pulsating colored light (Grote et al., 2013) can increase vmHRV when delivered at the frequency of 0.1 Hz. This effect is speculated to reflect a resonance in the cardiovascular system between the naturally occurring Meyer wave and the heart rate response to external/internal rhythmic stimulation. In such a case, the target of electrical stimulation would not be important at all, and the observed by Keute et al. (2021) increase in vmHRV would be a result of tactile stimulation at 0.1 Hz versus lack of stimulation in the control group. In the latest study that combined slow breathing with taVNS, slow-paced breathing was performed during both taVNS and the sham stimulation of the earlobe (Veiz et al., 2021). The study found neither a general effect of taVNS on vmHRV nor a facilitating effect of taVNS on vmHRV during slow breathing. However, the slow breathing task lasted only three minutes, and, in contrast to the stimulation at 0.1 Hz used by Keute et al. (2021), taVNS was delivered in a continuous fashion, increasing the possibility of adaptation to stimulation.

The present study aimed to examine the effects of taVNS combined with slow breathing on vmHRV and address some of the limitations of previous studies. In this study, we used a cross-over design to compare taVNS combined with slow breathing to slow breathing combined with sham stimulation on the earlobe. In contrast to the study by Keute et al. (2021), electric stimulation at the frequency of 0.1 Hz was present in both conditions, so the only difference between conditions was whether or not electrical stimulation occurred at a location where the vagus nerve innervates the ear’s skin (Peuker & Filler, 2002). In contrast to studies by Frøkjaer et al. (2016), and Juel et al. (2017) this design allowed us to partially disentangle the cardiac effects of slow breathing from the effects of taVNS, because slow breathing was also present in the control condition. Furthermore, in the present study, taVNS combined with slow breathing was performed for twenty minutes, a significantly longer duration than in the studies by Keute et al. (2021) and Veiz et al. (2021). This allowed the exploration of the potential role of the duration of stimulation (dosage) on cardiac effects.

In the present study, the stimulation was delivered during expiration because several authors have recently speculated that during spontaneous breathing taVNS can be optimized by delivering it during expiration, when cardio-respiratory sensory vagal pathways are most active (Garcia et al., 2017, 2021; Napadow et al., 2012; Sclocco et al., 2019) Slow breathing increases activation of the vagal interceptors that are activated during expiration, therefore it can be hypothesized that synchronization of taVNS with expiration during slow breathing may be more effective than delivering it during inspiration. Furthermore, the current study employed a stimulation frequency of 100 Hz, which deviates from the more commonly used frequency of 25 Hz in taVNS studies. Preliminary evidence showed that taVNS at 100 Hz results in greater effects than stimulation at lower frequencies (Sclocco et al., 2020; Stowell et al., 2019). Specifically, taVNS at 100 Hz, but not at 2, 10, or 25 Hz was found to effectively decrease blood pressure in hypertensive patients (Stowell et al., 2019). In another study using fMRI, stimulating at 100 Hz generated greater brainstem activations compared to 2, 10, and 25 Hz (Sclocco et al., 2020). Similarly, a recent study found that stimulation at 100 Hz was associated with a greater reduction in heart rate compared to lower frequencies (1, 10, 25 Hz; Yokota et al., 2022). Potential reasons for these observations are that different stimulation frequencies act upon different sensory receptor types (Pacinian corpuscles in the case of 100 Hz; Meissner corpuscles for low frequencies), and/or the frequency-dependent nature of brainstem response to signals from afferent vagus nerve pathways (Stowell et al., 2019).

## Method

### Participants

A total of 22 healthy students (15 women and 7 men) from the University of Warsaw between the ages of 19 and 29 years (*M* = 22.14, *SD* = 2.62) were recruited through an announcement via a social media outlet. Exclusion criteria were any chronic diseases, psychiatric and neurological disorders, cardiovascular diseases, respiratory diseases, and any implantable electronic devices. Participants were informed about exclusion criteria during recruitment. The sample size was computed using G*power 3.1 software (Faul et al., 2007). The analysis was conducted using one-tailed paired samples *t*-tests (for a comparison of differences between conditions in the change of vmHRV between baseline and taVNS/sham). Because the study was aimed at detecting clinically significant effects, the sample size was computed for a large effect size of: *d*_z_=0.8, an alpha of 0.05, and a power of 0.95. The analysis showed that the study required a sample of 19 participants. The sample size was increased to 22 to compensate for the potential removal of participants from the analysis. One participant did not come for the second laboratory session and one subject dropped out because the structure of their ear made it impossible to attach the electrode to the tragus, one participant had many ectopic beats, and three participants did not decrease respiratory rate during the slow breathing task, and were therefore removed from the analyses. Furthermore, due to device failure, one ECG was not recorded during the recovery period in one participant. In summary, the final HRV analysis was conducted on 15 participants (5 men and 10 women).

### Study Design

**Fig. 1:**
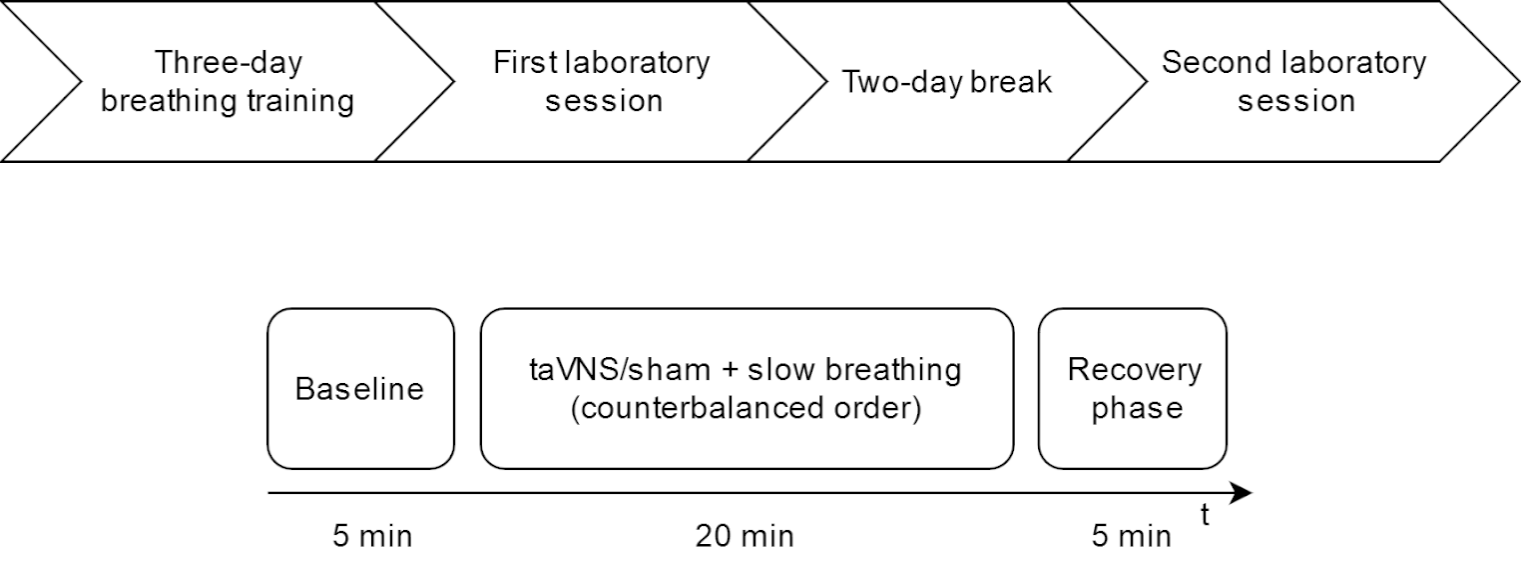
Study design (upper panel) and structure of one laboratory session (lower panel). The laboratory sessions differed only in the use of taVNS or sham stimulation

The experiment had a crossover design (see Fig. 1). Before the first laboratory session, participants performed a three-day online training of slow paced breathing to make the breathing task more comfortable and reduce hyperventilation. Next, each participant took part in two experimental sessions: one with taVNS combined with slow breathing, and another one with sham stimulation combined with slow breathing. The order of conditions was random and counterbalanced. Laboratory sessions were separated by two days. Because cardiovascular activity changes across the circadian cycle (Guo & Stein, 2003), each participant’s two sessions took place at the same time of the day (± 2 h). Participants were asked to refrain from intense physical activity on the day of laboratory measurement. Furthermore, they were asked to restrain from caffeinated beverages and smoking tobacco for four hours before the study. The study was approved by the ethical committee of the Department of Psychology, University of Warsaw.

### taVNS/Sham Stimulation and Paced Breathing

**Fig. 2:**
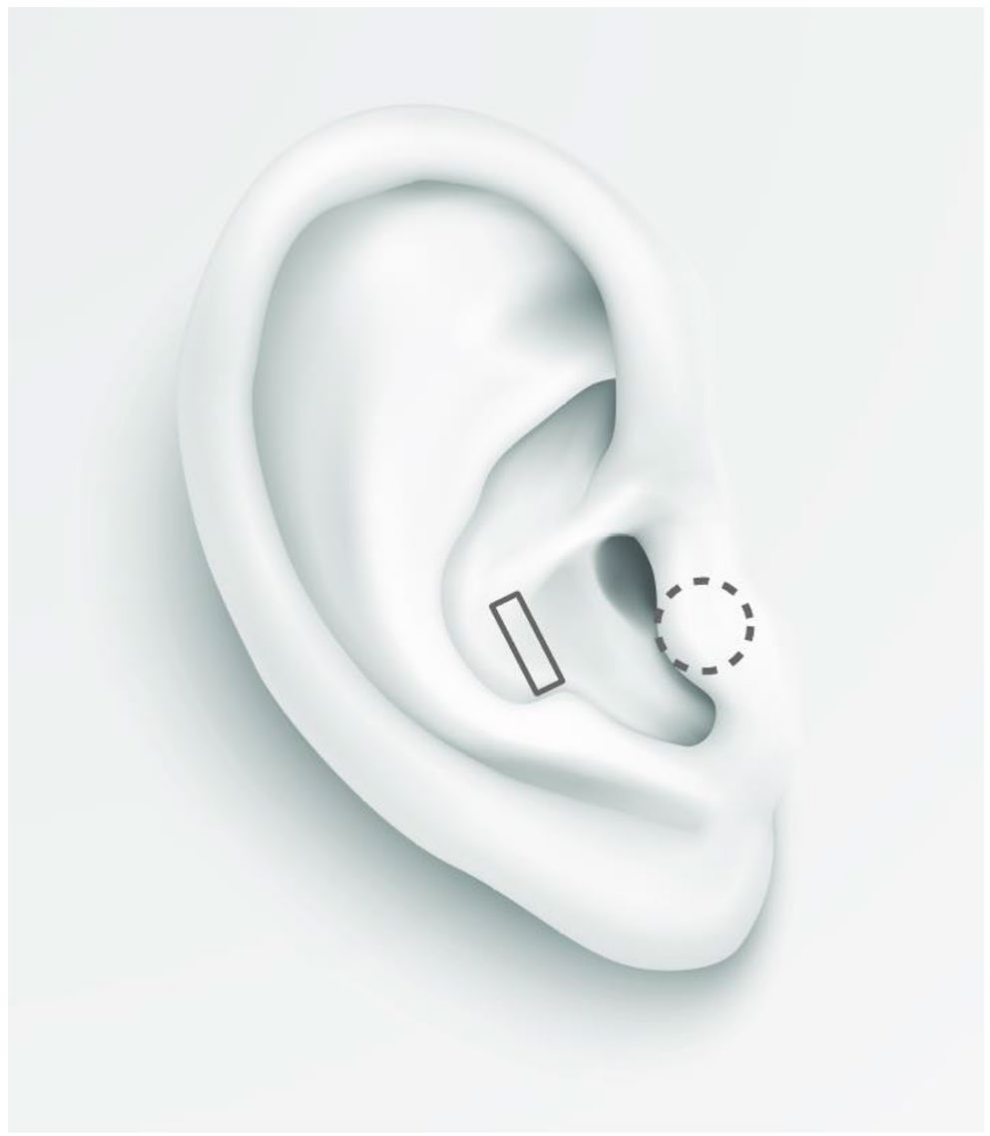
Location and shape of electrodes indicated by a gray rectangle and circle (dashed line indicates the placement of the electrode on the inside of the tragus)

**Fig. 3:**
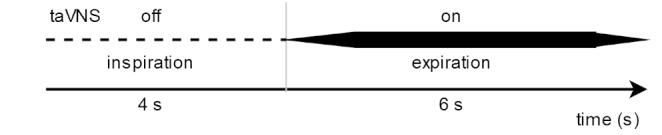
The synchronization of taVNS with the breathing phase and time of off (inspiration)/on (expiration) phases. The figure also displays the gradual increase and decrease of stimulation intensity during the first and last seconds of taVNS.

Electrical stimulation was delivered with the use of the transcutaneous electrical nerve stimulation device ECO 22 (ECONOVA, Szczecin, Poland). It is a constant voltage device that allows control of basic stimulation parameters. The device was set to produce biphasic symmetrical rectangular pulses with 400 µs duration and a frequency of 100 Hz. Two ear clip electrodes were placed on the left ear: one on the inner tragus and the second one on the cavum conchae (in the active condition), and both of them on the earlobe in sham condition (see Fig. 2). These electrode locations allowed the use of electrodes with larger surfaces than during stimulation of the cymba conchae. Thanks to this, the stimulation was perceptible enough to serve as a breathing pacer through 20 min of stimulation. Conductive electrode gel (Żelpol EKG; Centrum Medicum Poland, Łódź, Poland) was used to facilitate the delivery of electric stimulation. For each participant, the intensity of stimulation was calibrated by gradually increasing it to the point of the first painful intensity and then decreasing it to the intensity just below the pain threshold, so that the highest intensity was below the pain threshold. Stimulation intensity increased gradually for one second. Stimulation lasted for four seconds and gradually decreased for one second (see Fig. 3). Inter-stimulation intervals lasted 4 s. Thus, every cycle of stimulation lasted 10 s (see Fig. 3). Participants were asked to exhale during the stimulation, which means that participants breathed with a respiratory rate of 6 breaths per minute (0.1 Hz).

To improve the performance of the breathing task during the experiment, we employed a simple online slow-breathing training prior to the experiment. Paced breathing among untrained individuals can lead to hyperventilation and dyspnea (Szulczewski & Rynkiewicz, 2018). One recent study suggests that brief training of paced breathing decreases the tendency to hyperventilate and makes the task more comfortable and pleasant (Szulczewski, 2019a). Therefore, participants trained paced breathing individually for three days before the experiment. An audiovisual breathing pacer was provided via the internet, and participants accessed it with individual codes (to allow us to check when and how often they used the breathing pacer). Some participants showed a worse than intended adherence to this training, but this potential limitation can be expected to affect the (within subject) taVNS and sham conditions to a similar degree. The analysis therefore also included the data of the participants who did not perfectly adhered to the three-day pre-experimental training of slow breathing. Adherence to the training was as follows: 11 participants performed breathing practice three times, 2 participants did so twice, one participant accessed training one time, and one participant did not participate in training at all. Furthermore, to decrease hyperventilation, a short anti-hyperventilation instruction was provided before paced breathing tasks (Szulczewski, 2019b).

### Physiological Measures

Electrocardiogram data was recorded with a three-lead ECT‐3 amplifier (developed at the University of Groningen, Netherlands). Electrodes were placed on the chest: two on either side of the rib cage, and a ground electrode was placed on the sternum. The electrocardiograph (ECG) signal was sampled at 1000 Hz. Recordings were visually inspected in HEPLAB (Perakakis, 2019), corrected in ARTiiFACT with cubic spline interpolation, and exported as mean interbeat intervals (IBIs). Further analyses were done in Kubios 3.5.0 (Tarvainen et al., 2014). Mean IBI and vmHRV were computed for three periods: baseline, twenty minutes of vagal/sham stimulation, and recovery phase. The vmHRV was computed with a time-domain method (root-mean-square of successive differences; rMSSD). Furthermore, the amplitude of respiratory-related HR oscillations during the breathing task was assessed by the frequency method (fast Fourier transform) as the power spectrum (ms^2^) of the frequency band around the frequency of 0.1 Hz (0.9–0.11 Hz). Power values were log-transformed (log10) to obtain a normal distribution. Because vmHRV analysis is sensitive to the length of measurement (Shaffer & Ginsberg, 2017), analyses were first conducted for four five-minute periods of slow breathing, and then mean values for slow breathing were computed and used in further analyses. This allowed the comparison of twenty minutes of slow breathing to a five-minute baseline and recovery period. Blood pressure was measured with the Portapres Model 2 (Finapres Medical Systems, Enschede, Netherlands). However, data on blood pressure is not reported in this article because of the low quality of recordings and the large number of recordings that had to be removed from analysis due to excessive calibration and errors in measurements.

To check whether the targeted respiratory rate was reached during slow breathing, participants wore a nose cannula connected to an infrared sidestream capnometer (Capnocheck Plus, model 9004-000, BCI International, Waukesha, USA). The respiratory rate was estimated by the device’s internal algorithm and recorded on PC. Mean values for baseline, vagal stimulation, and recovery phase were computed in MATLAB (release 2014, The MathWorks, Natick, USA).

### Procedure

At the beginning of the first laboratory session, participants signed a consent form and were connected to the physiological devices (ECG electrodes, nasal cannula, and finger cuff). Each session began with a three minute pre-baseline period and a five minute baseline, during which participants were asked to sit with open eyes. Next, taVNS electrodes were attached to the ear and twenty minutes of taVNS/sham stimulation began. Participants were instructed to exhale during electrical stimulation and breathe only through their nose with opened eyes. After stimulation, participants were asked to sit with open eyes for the next five minutes (recovery phase). During the study, two dimensions of affective state were measured four times with the use of two sliders. However, because the sliders were implemented in a way that produced a frequent error in measurement, the self-reported data is not included in this article.

### Statistical Analysis

The analyses were conducted with JASP software (version 0.16, University of Amsterdam, Netherlands). For frequentist statistics, a significance threshold of *p* < .05 was used. Furthermore, Bayes factors (BFs) were computed for non-significant findings that were crucial for our hypotheses to investigate whether there is evidence in favor of *H*_0_ relative to *H*_1_. All Bayesian analyses were performed with default Cauchy priors. BFs were interpreted based on a classification scheme proposed by Jeffreys (Jarosz & Wiley, 2014). In order to check whether participants decreased respiratory rate during slow-paced breathing, we conducted a repeated-measures ANOVA with phase of the experiment (baseline, slow breathing, and recovery phase) and condition (taVNS and sham stimulation) as factors. Mauchly’s Tests of Sphericity were used to check for violation of the sphericity assumption (not violated for any variable) and Bonferroni correction was applied for posthoc tests for all ANOVAs for repeated measures. To further examine whether there was a difference between conditions in respiratory rate during slow breathing, a Bayesian paired sample *t*-test was conducted for the mean respiratory rates of both conditions. Also, stimulation intensity between conditions was compared with Bayesian paired sample *t*-tests. To examine changes in mean IBI and rMSSD, ANOVAs for repeated measures were conducted with phase of the experiment (baseline, slow breathing, and recovery phase) and condition (taVNS and sham stimulation) as factors. Next, Bayesian paired sample *t*-tests were conducted for the difference between slow breathing and baseline, as well as recovery phase and baseline in vmHRV and mean IBI of both conditions. Bayesian analyses were conducted assuming a lack of difference between conditions in respiratory rate (RR) and stimulation intensity (two-tailed tests), and larger vmHRV and mean IBI during slow breathing in conditions with taVNS in comparison to sham stimulation (one-tailed tests).

## Results

Means and standard deviations for each stage of the experiment are presented in Table 1. A repeated measures ANOVA conducted for respiratory rate indicated that RR changed during the experiment, *F*(2, 22) = 41.31, *p* < .001, *η*^*2*^ = 0.58. Post-hoc tests revealed that RR was lower during slow breathing than during baseline (*p* < .001) and the recovery phase (*p* < .001). Analysis showed a non-significant interaction effect between the moment of measurement and condition, *F*(2, 22) = 1.77, *p* = .19, *η*^*2*^ = 0.02. A Bayesian paired sample *t*-test for RR during slow breathing provided anecdotal evidence for *H*_0_ relative to *H*_1_, *BF*_*01*_ *=* 2.35, and error % = 0.02. Analysis of stimulation intensity (mean intensity on the device’s scale: taVNS = 3,68; sham = 3,5) yielded *BF*_*01*_ *=* 3.57, and error % = 0.00, indicating substantial evidence for *H*_0_ relative to *H*_1_. Thus, analyses showed that participants’ respiratory rates decreased and that conditions were comparable in terms of both respiratory rate during slow breathing as well as stimulation intensity.

**Table 1:** Means and standard deviations (in brackets) for each stage of the experiment and both conditions

	taVNS			Sham stimulation		
Measure	Baseline	Stimulation	Recovery	Baseline	Stimulation	Recovery
RR	12.33 (3.19)	6.94 (1.06)	12.28 (2.66)	13.12 (3.83)	6.73 (0.80)	10.69 (2.78)
IBI	780.66 (104.88)	802.46 (79.92)	802.14 (83.86)	796.67 (108.70)	824.25 (106.71)	839.92 (106.03)
rMSSD	38.48 (16.66)	53.91 (23.66)	39.87 (12.32)	40.76 (21.63)	57.20 (25.41)	48.87 (26.53)
0.1 power (log10)	-	3.72 (0.28)	-	-	3.69 (0.33)	-

Notes. RR = respiratory rate; IBI = mean interbeat interval; rMSSD = root mean square of successive differences between normal heartbeats; 0.1 power (log10) = the power spectrum (ms2) of the frequency band of 0.9–0.11 Hz

A repeated measures ANOVA conducted for mean IBI showed a main effect of the phase of the experiment, *F*(2, 26) = 7.15, *p* < .01, *η*^*2*^ = 0.05, and post-hoc analysis revealed that mean IBI increased from baseline to the slow breathing (*p* < .05) and the recovery phases (*p* < .01). The interaction between the phase of the experiment and condition was non-significant, *F*(2, 26) = 0.74, *p* = .47, *η*^*2*^ = 0.00, suggesting a lack of effect of taVNS in comparison to sham on heart rate. Next, to examine the strength of the evidence for the lack of effect of taVNS on mean IBI, a Bayesian paired sample *t*-test was conducted for the differences between slow breathing and baseline, as well as recovery and baseline, for both conditions. Comparison of differences between slow breathing and baseline yielded substantial evidence for *H*_0_ relative to *H*_1_, *BF*_*01*_ *=* 5.39, and error % = 0.00, as well as for differences between recovery phase and baseline, *BF*_*01*_ *=* 7.38, and error % = 0.00. Therefore, analyses showed that mean IBI increased during the experiment (meaning that heart rate decreased), but there were no differences between conditions in the magnitude of heart rate deceleration.

**Fig. 4:**
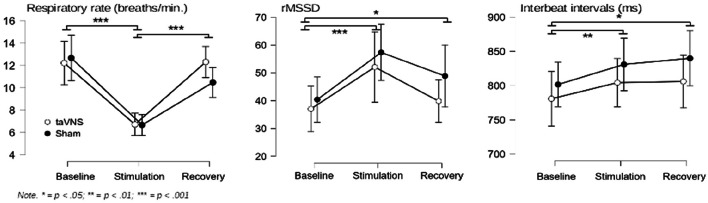
Mean respiratory rate, root-mean-square of successive differences (rMSSD), and interbeat interval (IBI) for the three timepoints of the study and both conditions (separate lines), along with 95% confidence intervals. Asterisks indicate the significance of the effect of the phase of the study (baseline, stimulation, and recovery). All interactions between phase and condition were non-significant

A repeated measures ANOVA conducted for rMSSD showed a main effect of the phase of the experiment, *F*(2, 26) = 17.94, *p* < .001, *η*^*2*^ = 0.16. Post-hoc analysis showed that rMSSD increased during the slow breathing (*p* < .001), and recovery phases (*p* < .05) relative to baseline. The interaction between phase and condition was non-significant, *F*(2, 26) = 1.00, *p* = .38, *η*^*2*^ = 0.00. To further examine this null finding we performed a Bayesian paired sample *t*-test comparing the differences between slow breathing and baseline, as well recovery and baseline, for both conditions. Analyses provided substantial evidence for *H*_0_ relative to *H*_1_, for both changes between baseline and slow breathing combined with taVNS/sham, *BF*_*01*_ *=* 4.45, and error % = 0.00, as well as the recovery phase, *BF*_*01*_ *=* 8.61, and error % = 0.00. Next, a Bayesian paired sample *t*-test was performed to compare the power of heart rate oscillations around breathing frequency (0.1 Hz). This test provided anecdotal evidence for *H*_0_ relative to *H*_1_, *BF*_*01*_ *=* 2.98, and error % = 0.01. A graph showing the changes in physiological parameters during the experiment can be found below (Fig. 4). To sum up, our analyses showed that rMSSD increased during slow breathing, but no differences were found between taVNS and sham stimulation in the magnitude of the rMSSD increase. Furthermore, the analysis suggested that there was no difference in the amplitude of respiratory-related heart rate oscillations during slow breathing.

## Discussion

The present study investigated the effects of slow breathing combined with transcutaneous auricular vagus nerve stimulation (taVNS) on vagally mediated heart rate variability (vmHRV). In both conditions vmHRV increased. Participants performed slow breathing during both taVNS and sham stimulation (delivered to the earlobe). The observed lack of difference between conditions suggests that there is no acute effect of taVNS on vmHRV when applied during slow breathing. This finding is consistent with the results of a recent Bayesian meta-analysis that provided strong evidence for no immediate effects of taVNS on vagal heart control measured by analysis of vmHRV (Wolf et al., 2021). This meta-analysis included mostly studies that did not combine taVNS and slow breathing. Because slow breathing modulates the activity of cardio-respiratory vagal afferents, the present study was based on the hypothesis that the effects of slow breathing on vmHRV can be augmented by synergy with taVNS (Frøkjaer et al., 2016; Szulczewski, 2022). The lack of effect of slow breathing combined with taVNS in comparison to slow breathing with sham stimulation suggests that acute additive or synergistic effects are not present. The present study had a small sample size that was sufficiently sensitive only to detect large effects that would have clinical significance. Therefore, the study was biased towards the null hypothesis. However, the observed pattern of changes in vmHRV in both conditions does not suggest the existence of an effect that was missed because of insufficient statistical power (see Table 1 for mean values of root-mean-square of successive differences).

Previous research that combined taVNS with slow breathing and measured its effects on cardiovascular activity have provided mixed results. The first two studies that examined the effects of this combination compared it to sham stimulation with spontaneous breathing (Frøkjaer et al., 2016; Juel et al., 2017). Slow breathing produces a well-established and relatively large increase in indices of vmHRV and the use of a control group with spontaneous breathing made it impossible to draw conclusions about the contribution of taVNS to the observed effect. In the present study, we observed a lack of difference between slow breathing combined with taVNS versus sham stimulation. Therefore our findings suggest that the increase in indices of vmHRV observed by Frøkjaer et al. (2016) and Juel et al. (2017) was due to slow breathing rather than taVNS. In turn, a study by Keute et al. (2021) compared slow breathing combined with taVNS to slow breathing with a lack of electrical stimulation and observed increased vmHRV during taVNS. Our results suggest that the increase in vmHRV observed by Keute et al. (2021) was not caused by taVNS. Because previous studies showed that a variety of stimuli presented at 0.1 Hz may affect the amplitude of cardiovascular oscillations (Grote et al., 2013; Lehrer et al., 2009; Vaschillo et al., 2008, 2011), one possible explanation of the increased vmHRV in the study of Keute et al. (2021) is that it was the result of tactile stimulation delivered at the frequency of the Meyer wave (0.1 Hz) in comparison to lack any of stimulation in the sham condition. The present study supports the findings of Veiz et al. (2021) who reported a lack of acute effects of taVNS combined with three minutes of slow breathing. To sum up, the present findings suggest a lack of effect of taVNS on vmHRV during slow breathing. However, because studies differed in terms of stimulation parameters and location, it is possible that such differences are responsible for the heterogeneity in results.

It is believed that the effects of taVNS depend on stimulation parameters (Farmer et al., 2020). The previous studies that combined taVNS with slow breathing were heterogeneous in terms of stimulation parameters. The present study used a stimulation frequency of 100 Hz because it has been recently shown to produce a larger response in nucleus tractus solitarii (Sclocco et al., 2020) and in one study it was the only frequency effective at decreasing blood pressure (Stowell et al., 2019). In contrast, the study of Keute et al. (2021) applied an unusual burst stimulation pattern, with five 1 Hz bursts and an intra-burst frequency of 25 Hz. It can be hypothesized that this difference in stimulation parameters accounts for the observed differences in results. Interestingly, one recent study compared taVNS delivered in burst stimulation to tonic stimulation and observed a larger increase in vmHRV during burst stimulation (Shen et al., 2021). Therefore, optimization of the pattern of taVNS delivery seems to be an interesting direction for future research aimed at modulating cardiac vagal motor output.

Another potentially important parameter is the intensity of stimulation. Some previous studies that compared different stimulation intensities within-subjects suggested that stimulation at higher intensities may be more effective in modulating HRV (Machetanz et al., 2021; Yokota et al., 2022). However, a series of three, well-powered studies by Borges et al. (2019) could not confirm these results, and these null-findings were supported by Bayesian analyses. The current evidence remains inconclusive about whether and when stimulation intensity affects cardiac activity, because studies typically differ also in other respects. As an example, most studies use voltage- rather than current-controlled stimulation, which can result in a different amount of current being delivered to the tissue depending on the type of electrodes used (and their resistance), the amount and type of gel, etc. making comparison between multiple studies useless (Farmer et al., 2020). In addition, the calibration method used could potentially increase variability in physical stimulation intensity, as pain threshold varies between participants and is also variable within individual participants. Importantly, our findings did not indicate a different physical stimulation intensity between conditions in the current study, so physical stimulation intensity should not have confounded any observed effects of condition. Further research may want to address the yet still open question of whether using a fixed physical stimulation intensity in every participant and every condition would yield different findings.

The location of stimulation is another important parameter that is considered potentially relevant for the effects of taVNS (Yakunina et al., 2017). In the present study, taVNS was delivered to a large area of the ear in the regions that are not solely innervated by the auricular branch of the vagus nerve. One electrode was placed on the inner tragus and the second one on the cavum conchae. Apart from the vagus nervus, both of these regions are also innervated by the great auricular nerve, and the tragus is also innervated by the auriculotemporal nerve (Peuker & Filler, 2002). Furthermore, it can be speculated that current can spread from the tragus to adjacent areas (Kreisberg et al., 2021), which are innervated by the mandibular branch of the trigeminal nerve. This could potentially activate cardiac trigeminal reflex and potentiate cardiac effects of stimulation (Meuwly et al., 2015). However, the observed lack of effect on vmHRV suggests that activation of the trigeminal reflex did not occur. Previous studies that combined taVNS with slow breathing used stimulation of various ear regions. Frøkjaer et al. (2016) and Juel et al. (2017) stimulated the cymba conchae, which is believed to be innervated solely by the vagus nerve (Peuker & Filler, 2002). In contrast, Veiz et al. (2021) delivered stimulation to the tragus and Keute et al. (2021) compared both locations of stimulation, reporting a lack of difference in vmHRV between them. This finding is consistent with a recent study by Borges et al. (2021) that found no difference in vmHRV between stimulation of the cymba conchae and the tragus. However, a recent study by Machetanz et al. (2021) showed larger effects on vmHRV during stimulation of the cymba concha compared to the tragus. Thus, findings are inconsistent and do not provide a clear picture of the potential effects of the stimulation site on the effects of taVNS on vmHRV.

The last decade has seen an increase in research on taVNS, making it challenging to conduct a thorough review of existing studies. In this situation, meta-analytical systematic reviews are key to drawing firm conclusions. Currently, many studies that have obtained positive results present the modulation of the vmHRV by taVNS as a fact, but such a conclusion may be due to the selective and biased review of previous studies. This bias often limits discussion of differences between studies that could potentially account for the observed heterogeneity in results. Considering the strong evidence for the lack of effects obtained by a recent meta-analysis (Wolf et al., 2021), we strongly advocate tempering the certainty and generalizability of conclusions based on the relatively rare positive findings. Modulation of vagal motor control of the heart by taVNS would have many potential applications, which may further promote biases in the field.

Currently, the field of taVNS research is dominated by studies on the immediate effects of stimulation, but their absence does not rule out the possibility that an increase in vmHRV may occur as a result of long-term use of taVNS. This effect could be mediated by, for example, improved emotional state or decreased inflammation, because both negative affect disorders (e.g., anxiety and depressive disorders) and high levels of inflammation are related to low vmHRV (Michopoulos et al., 2017; Williams et al., 2019; Young et al., 2014).

The results of the present study do not support the idea that taVNS can be used to increase the cardiovascular effects of slow breathing. However, the current zero-finding regarding vmHRV does not rule out the combination of taVNS with slow breathing as a promising method to modulate other target systems for which both slow breathing and taVNS have been considered separately, such as pain and affective systems (for comprehensive reviews see: (Frangos et al., 2017; Jafari et al., 2017; Lehrer et al., 2020; Szulczewski, 2022; Zaccaro et al., 2018). Therefore, future studies may look for potential additive or synergistic effects of taVNS and slow breathing for targets other than vagal motor activity.
